# Letter from Dr. Talbot

**Published:** 1905-02

**Authors:** Eugene S. Talbot

**Affiliations:** Chicago


### Domestic Correspondence.

### LETTER FROM DR. TALBOT.

CHICAGO, December 14, 1904.

TO THE EDITOR:

SIR,—In the November INTERNATIONAL DENTAL JOURNAL, under "Bibliography," page 895, in reviewing Dr. Otto E. Inglis's work on "Dental Pathology and Therapeutics," the reviewer says, "The editor separates 'interstitial gingivitis,' as named by Talbot, from pyorrhœa alveolaris, making it a distinct disease. This is not as Talbot intended, for he distinctly renamed pyorrhœa alveolaris by this term, and it is difficult to understand how any pathologic distinctions can be made between these two as described by the editor."

In the same journal, on page 866, "Reports of Society Meetings," Academy of Stomatology of Philadelphia, appears the following:

"*Dr. M. Schamberg.*—There is a case which should be reported by Dr. Fogg. He brought to my office about a week ago a patient who had rather a peculiar condition upon the root of a central incisor tooth. He noticed a swelling upon the gum overlying the root of that tooth, and upon testing it found it to be vital and decided that the condition must be a pericemental abscess. He was very anxious to find some cause for the trouble, and a radiograph was taken which showed a large area of tooth destruction. This destruction indicated resorption. The tooth was kept under observation for quite a while. Yesterday, the doctor told me that, in addition to this tooth, quite a number of the other teeth exhibited this irregular resorption, to such an extent, indeed, that the tooth-crown was lost. It is the only case of the kind I have ever seen, and it is of such an unusual type that I thought it might be interesting for the Academy to hear of the case. I would be very glad to know whether any have met with a similar condition.

"*Dr. E. T. Darby.*—Were they all vital teeth?

"*Dr. Schamberg.*—They were in the main vital. In some of the lower teeth the pulps were dead.

"*Dr. James Truman.*—How old was the patient?

"*Dr. Schamberg.*—The patient was about thirty-two or thirty-five years old.

"*Dr. H. R. D. Swing.*—Was there any pyorrhœa?

"*Dr. Schamberg.*—There was no pyorrhœa, but there was a certain amount of recession. The tooth was vital up to the time of its removal.

"*Dr. I. N. Broomell.*—Had the alveolar bone filled in the space?

"*Dr. Schamberg.*—No; the alveolar process in most cases was resorbed over the site of the root resorption, due more to infection which reached the part in consequence of the food débris entering the spaces. Almost every surface of the molar teeth affected showed resorption."

The two quotations bring into view the entire pathology of the alveolar process. There seems yet mystification as to etiology, progress, and termination of the inflammation of the alveolar process. Dr. Inglis's arrangement is an improvement over other works on pathology. There was no intention on my part to "rename" as "interstitial gingivitis" "pyorrhœa alveolaris," except as this term is loosely and popularly used to indicate a specific disorder. Pyorrhœa alveolaris is the result of disease, and therefore a secondary state. Interstitial gingivitis is a disease, just as interstitial nephritis is a disease, which affects the deeper structures of the kidney. If the disease be due to local causes, such as tartar, a tooth cavity, unfinished fillings, etc., there is, first, gingivitis, then interstitial gingivitis, and last pus infection or pyorrhœa alveolaris. If pus germs occur in the proximity, the part may become infected at either stage of the disease. The patient from pregnancy, or from drug poison, scurvy, or auto-intoxication, may and frequently does have interstitial gingivitis. The gingivitis is secondary to the interstitial stage because the deeper structures are first involved. This is due to the exceedingly transitory nature of the alveolar process and to the alveolar process holding the roots of the teeth (virtually foreign bodies), thus being an end organ. In the second expression of the disease there generally is the infection constituting pyorrhœa alveolaris.

Employment of the term pyorrhœa alveolaris, except where pus flows from the sockets or gums, is not justifiable. The term should be restricted to its proper place and proper time by teachers of pathology. Interstitial gingivitis is an inflammation of the alveolar process which attacks every mouth after the alveolar process has obtained its growth. In a majority of cases there is no gingivitis. Absorption of the alveolar process begins as soon as the building up process ceases. When a tooth erupts, there is interstitial gingivitis. When a tooth is extracted, absorption is produced by interstitial gingivitis. When a patient becomes infected by syphilis or gonorrhœal germs, interstitial gingivitis occurs and the teeth become more or less loose. Underlying the trophic tooth and jaw changes of the great neuroses, tabes dorsalis, paretic dementia, etc., is interstitial gingivitis.

Change of climate and food, with slow adjustment of the organs of the body, as observed in the English soldiers and women and children in the concentration camps of South Africa, American soldiers in Cuba and the Philippines, miners in the Klondike, and men working on the Jungfrau Railroad in Switzerland, produce interstitial gingivitis.

The best illustration of interstitial gingivitis is the inflammation of the peridental membrane and alveolar process in the formation of an alveolar abscess. All inflammation of the alveolar process from any cause is an interstitial gingivitis.

Since every one has potential interstitial gingivitis after the alveolar process has obtained its growth, the severity of the disease depends upon the etiologic moment. Only a very small per cent., say five, has the secondary symptoms, pus infection.

The nature of the case of Dr. Schamberg, quoted above, is now clearer. This patient was the victim of extreme interstitial gingivitis, not pyorrhœa, just as Dr. Schamberg stated. This was due to some systemic condition which skiagraphy would not solve. Osteoblasts and osteoclasts build up or tear down tooth or bone structure by a process of inflammation. Here, as in pseudohypertrophic paralysis, two opposite processes arise from the same inflammatory process. Exostosis and root absorption are liable to occur. Since the cause of the interstitial gingivitis is systemic or constitutional, we would expect all the teeth to be absorbed more or less. It is also to be expected that the alveolar process is absorbed about the roots, if not entirely, not from "infection" due to "food débris," but from interstitial gingivitis.

In the same manner absorption of the temporary teeth occurs. The permanent crowns start to erupt. They set up an interstitial gingivitis. The interstitial gingivitis sets the osteoclasts at work, and the roots of the temporary teeth are thus removed. The question naturally arises, Why are not the roots of the temporary teeth whose pulps are destroyed absorbed? Occasionally they are; now only a small portion, then in most cases not at all. The reason is that irritation and infection produced by the decomposition of the dead pulp is so severe that interstitial gingivitis is extensive. Not only has an alveolar abscess formed, but bone absorption area is quite large. The trabeculæ are thus destroyed and the osteoclasts deprived of a resting-place near the root, hence absorption is impossible. The term pericemental is used. Here a proper knowledge of pathology is wanting. Since interstitial gingivitis is, as a rule, general and not circumscribed, the abscess may be located at any point where the infection is the greatest. I have demonstrated these points of infection and abscess on the outer surface of the alveolar process as well as at the centre. The term "peridental," therefore, is more to the point than "pericemental."

EUGENE S. TALBOT.

[Dr. Talbot's assertion in the foregoing, that "There was no intention, on my part, to 'rename' as interstitial gingivitis, pyorrhœa alveolaris," is somewhat at variance with his published work. The reviewer of the Burchard-Inglis "Dental Pathology" accepted Dr. Talbot's statements on the title-page of his book and in the introduction as conclusive. He names his work "Interstitial Gingivitis, or So-called Pyorrhœa Alveolaris," and in the "Introduction" he says, "With a view of clearing up this question at the outset by the use of a proper title, I have adopted as a designation for the condition hitherto known as pyorrhœa alveolaris, the term "Interstitial Gingivitis." It seems impossible to give any other meaning to these quotations except that it was the expressed intention to rename this pathological condition. The frequent use of it by writers in place of the old term seems to accentuate the position taken by the reviewer.—EDITOR.]

